# *Solanum malmeanum*, a promising wild relative for potato breeding

**DOI:** 10.3389/fpls.2022.1046702

**Published:** 2023-02-20

**Authors:** Rodrigo Nicolao, Paola Gaiero, Caroline M. Castro, Gustavo Heiden

**Affiliations:** ^1^ Programa de Pós-Graduação em Agronomia/Fitomelhoramento - Universidade Federal de Pelotas (UFPel), Pelotas, RS, Brazil; ^2^ Departamento de Biología Vegetal, Facultad de Agronomía, Universidad de la República, Montevideo, Uruguay; ^3^ Laboratório de Recursos Genéticos, Embrapa Clima Temperado, Pelotas, RS, Brazil

**Keywords:** conservation, crop wild relatives, food security, genetic resources, genebank, germplasm, *Petota*, Solanaceae

## Abstract

Crop wild relatives are gaining increasing attention. Their use in plant breeding is essential to broaden the genetic basis of crops as well as to meet industrial demands, for global food security and sustainable production. *Solanum malmeanum* (*Solanum* sect. *Petota*, Solanaceae) is a wild relative of potatoes (*S. tuberosum*) from Southern South America, occurring in Argentina, Brazil, Paraguay and Uruguay. This wild potato has been largely mistaken for or historically considered as conspecific with *S. commersonii*. Recently, it was reinstated at the species level. Retrieving information on its traits and applied uses is challenging, because the species name has not always been applied correctly and also because species circumscriptions and morphological criteria applied to recognize it have not been consistent. To overcome these difficulties, we performed a thorough literature reference survey, herbaria specimens’ identification revision and genebank database queries to review and update the information available on this potato wild relative, contributing to an increase in research on it to fully understand and explore its potential for potato breeding. Scarce studies have been carried out concerning its reproductive biology, resistance against pests and diseases as well as tolerance to abiotic stresses and evaluation of quality traits. The scattered information available makes it less represented in genebanks and genetic studies are missing. We compile, update and present available information for *S. malmeanum* on taxonomy, geographical distribution, ecology, reproductive biology, relationship with its closest relatives, biotic and abiotic stresses resistance and quality traits and discuss ways to overcome sexual barriers of hybridization and future perspectives for its use in potato breeding. As a final remark, we highlight that this species’ potential uses have been neglected and must be unlocked. Thus, further studies on morphological and genetic variability with molecular tools are fundamental for an efficient conservation and applied use of this promising genetic resource.

## 1 Introduction

Potato (*Solanum tuberosum* L.) is the third most important food crop in terms of global food security, after rice and wheat and the most important non-grain food crop (Birch et al., 2012). It is a prominent source of calories, fibers, proteins, mineral micronutrients, vitamins B6 and C, potassium, and antioxidant compounds, and can pack all those nutrients while using less area (m²) than any other crop (Birch et al., 2012; Devaux et al., 2014). Conventional potato cultivars are bred at the tetraploid level, have high heterozygosity, reproduce vegetatively and have less genetic variation than their wild relatives (Fu et al., 2009; Hardigan et al., 2015; Kolech et al., 2016; Hardigan et al., 2017; Duan et al., 2019; Drapal et al., 2020). Climate change and the dispersion of pests and new races of pathogens are currently a matter of concern among potato breeders and farmers. The outbreaks caused by new isolates of the late blight oomycete *Phytophthora infestans* (Mont.) de Bary in Europe and the Americas (Santana et al., 2013; Göre et al., 2019; Maurice et al., 2019), or by the new sequevars of bacterial wilt *Ralstonia solanacearum* found in Brazil, Peru and Iran (Gutarra et al., 2017; Santiago et al., 2017; Sedighian et al., 2020) have the potential to destroy potato fields. Lack of resistance of the current potato cultivars to the main diseases that affect the crop makes it one of the most dependent crops on agrochemical applications (Vleeshouwers et al., 2011). This scenario requires genetic variability for the continuous process of crop improvement to face biotic stressors and climate change, while increasing productivity to ensure global food security.

Crop Wild Relatives (CWR) are a reservoir of valuable genetic diversity for crop improvement, especially those genes that confer resistance to pests and diseases, tolerance to abiotic stressors and climate change scenarios, as well as providing nutritional compounds (Maxted et al., 2006; Jansky et al., 2013; Zhang et al., 2017; Pradheep et al., 2019; Perrino and Perrino, 2020). CWR contain pharmaceutical and industrial properties and can potentially contribute for a sustainable and smarter agroecosystem way (Jansky et al., 2013; Dempewolf et al., 2014; Warschefsky et al., 2014).

The cultivated potato has more than 100 wild relative species (*Solanum* sect. *Petota*, Solanaceae) (Douches et al., 1989; Hawkes, 1990; Pavek and Corsini, 2001; Spooner et al., 2014; Hardigan et al., 2017). Potato wild relatives (PWR) are widely distributed across the Americas, from the geographical and ecological points of view, ranging from Southwestern United States (38°N), through the dry areas of Mexico, until the adjacent mainland areas of Chile (41°S) in the Southern Cone of South America, at elevation ranging from sea level up to 4,500 m (Hijmans and Spooner, 2001; Spooner et al., 2004; Spooner et al., 2014; Spooner et al., 2016; Spooner et al., 2019). About 70% of these species are diploids while autopolyploids and allopolyploids go up to 6x chromosome numbers (x=12). PWR possess a common (basic) A genome, which, modified to different degrees, originated four additional genomes: B, C, D and E (Matsubayashi, 1991). Furthermore, the differentiation in the basic A genome is assumed to play a minor role as an isolation mechanism (Camadro et al., 2004).

To guide potato breeders in the efficient use of PWR according to their gene pool level, Spooner and collaborators (2014) proposed five crossability groups, based on the Endosperm Balance Number (EBN) along the ploidy, and self-compatible/self-incompatible systems. Diploid potato species (1EBN and 2EBN) are mostly self-incompatible (Cipar et al., 1964; Spooner et al., 2014). All 4EBN potato species are self-compatible due to a lack of pre-zygotic gametophytic barriers (Spooner et al., 2014). A self-compatible plant of *S. chacoense* was found (Hanneman, 1985) and the responsible *S-loci-inhibitor* (*Sli*) gene was identified and mapped to chromosome 12 (Hosaka and Hanneman, 1998a; b). The EBN post-zygotic barrier of hybridization acts at the embryo-endosperm development level, leading to aberrations in chromosome pairing and is observed through embryonic abortion, male sterility and breakdown of F1 or F2 hybrid offspring (Camadro et al., 2004; Camadro et al., 2012). According to Johnston et al. (1980), this post-zygotic barrier is explained under the hypothesis of the Endosperm Balance Number (EBN), in which *Solanum* sect. *Petota* species have a true ploidy number (determined by their actual number of chromosomes) and an “effective” ploidy generated by the action of hypothetical genetic factors (called EBN) (Johnston et al., 1980). The EBN system requires a 2:1 maternal to paternal EBN dosage for a normal endosperm development, which could predict the success of hybridization of some specific crosses. EBN is under genetic control (Ehlenfeldt and Hanneman, 1988; Camadro and Mansuelli, 1995; Johnston and Hanneman, 1996) and its values have been determined empirically for most PWR species by crossing each one with a standard species of known EBN (Hanneman, 1994; Mansuelli and Camadro, 1997). Due to the strong post-zygotic barrier of EBN differences, 2x (1EBN) species are sexually isolated from 2x (2EBN) and both 4x (4EBN) (Camadro and Peloquin, 1981; Hanneman, 1994).

*Solanum malmeanum* Bitter (Solanaceae) is a 1EBN PWR species, belonging to *Solanum* sect. *Petota* Dumort. ser. *Commersoniana* Buk., classified into the tertiary gene pool of potato (*S. tuberosum* L.) (Hanneman, 1994; Spooner et al., 2014). *Solanum malmeanum* is native to the Southern Cone of South America, occurring in Argentina, Brazil, Paraguay and Uruguay. This PWR was collected for the first time in 1893 by the Swedish botanist Gustaf Oskar Andersson Malme along the Ijuí river, at Rio Grande do Sul state in Southern Brazil (53° 54’ 53” W, 28° 23’ 16” S) and the new species was named after him when formally described later by the German botanist Friedrich August Georg Bitter in 1913 (Bitter, 1913). Taxonomic treatments of PWR by Bitter (1913); Correll (1962); Hawkes and Hjerting (1969); Mentz and Oliveira (2004); Matesevach and Barboza (2005), and Spooner et al. (2016) based on morphological traits, considered *S. malmeanum* as *S. malmeanum*, *S. commersonii* f. *malmeanum*, *S. commersonii* subsp. *malmeanum*, *S. commersonii* f. *malmeanum*, *S. commersonii* subsp. *malmeanum*, and *S. malmeanum*, respectively. *Solanum malmeanum* is a long day adapted PWR, highly flowering and a good pollen producer, having a geographical distribution that partially overlaps with *S. chacoense* Bitter and *S. commersonii* Dunal (Correll, 1962; Hawkes and Hjerting, 1969; Spooner et al., 2016). After a long history of being considered conspecific with *S. commersonii*, recently Spooner et al. (2014); Spooner et al. (2016) have reinstated the taxon at species level.

*Solanum malmeanum* populations have co-evolved with many diverse environmental conditions, including humid tropical and subtropical areas, semidesert conditions (e.g., dry, cold, frost, heat, salinity) and biotic stressors (Hawkes, 1958; Hawkes and Hjerting, 1969). Therefore, *S. malmeanum* stands out as a promising important source of allelic diversity and valuable agronomic traits for potato breeding, including resistance to pests and diseases, abiotic stress tolerance and industrial traits (Douches et al., 1989; Hanneman, 1989; Flanders and Radcliffe, 1992; Spooner and Bamberg, 1994; Jansky, 2000; Siri et al., 2009; Jansky et al., 2013; Bachmann-Pfabe and Dehmer, 2020).

Due to a changing taxonomic history, several morphological shared traits and geographical distribution patterns that greatly overlap, *S. malmeanum* has been largely confused or historically considered as conspecific with *S. commersonii*. Nevertheless, it was reinstated at the species level (Spooner et al., 2016). Due to the instability of the cohesive application of this species name and discordant species circumscriptions and morphological borders applied to recognize it along the taxonomic history, retrieving information on its traits and applied uses is tricky. To overcome these difficulties, we performed a thorough literature review, genebank database queries and a revision of herbaria voucher specimens cited in published studies to update the information available on this wild potato relative, aiming to broadening the scientific knowledge on the species to fully understand and explore its potential for potato breeding. Thus, our study aims to compile, synthetize and update all the scattered information about reproductive biology and cytogenetics, taxonomic treatments and geographical data, biotic and abiotic resistance of *S. malmeanum*, providing it in an elucidative and comprehensive way.

## 2 Materials and methods

### 2.1 Species circumscription

Our circumscription of *S. malmeanum* is based on the species original publication by Bitter (1913), the revised taxonomic treatment of Spooner et al. (2016); Nicolao (2021) and complementary observations from characterized genebank accessions available at database AleloVegetal (https://av.cenargen.embrapa.br/avconsulta/Home/index.do). When the original data discussed in this review was formerly published as *S. commersonii*, but the cited accession or specimen corresponds to *S. malmeanum*, we added the remark “as *S. commersonii*” right after.

### 2.2 Data review

To perform this review, we searched for “*Solanum commersonii*”, “*Solanum malmeanum*”, “*Solanum commersonii* subsp. *malmeanum*”, “*Solanum commersonii* f. *malmeanum*” and their abbreviated forms in literature, herbaria and genebank databases as follows.

### 2.3 Literature consulted

We reviewed the monographs for taxonomic treatments of tuber-bearing *Solanum* species conducted by Bitter (1913); Correll (1962); Hawkes and Hjerting (1969), and Spooner et al. (2016), as well as regional taxonomic studies for Argentina by Matesevach and Barbosa (2005), South of Brazil by Mentz and Oliveira (2004) and Paraguay by De Egea et al. (2018). The scientific reports published until October 31^st^ 2022 were searched in the databases Base de Dados da Pesquisa Agropecuária (BDPA 2022), Biblioteca Digital Brasileira de Teses e Dissertações (BDBTD 2022), CAPES Portal de Periódicos da Coordenação de Aperfeiçoamento de Pessoal de Nível Superior (2022), Google Scholar (2022), JSTOR (2022), Scientific Electronic Library Online (SciELO 2022), ScienceDirect system (2022), and printed publications available at the Embrapa Clima Temperado library.

### 2.4 Taxonomic and geographical distribution data

To update the taxonomic and geographic data for *S. malmeanum*, we reviewed, either through specimens loans or online, the label data of specimens from the herbaria B, BAL, BHCB, BM, CEN, ECT, F, ICN, K, MVFA, MVJB, MVM, MO, NY, P, R, RB, S, SPF, U, and US (acronyms according to Thiers 2022). Georeferenced records were extracted from the online platform databases Global Biodiversity Information Facility, speciesLink. Duplicate records were removed. Manual checking and correction of wrong or doubtful coordinates were carried out in GEOlocate (http://www.geo-locate.org/), Google Earth and Google Maps. The occurrence data were plotted on a map rendered in QGIS (2022).

### 2.5 Genebank databases search

To assess the representativeness of *S. malmeanum* in the germplasm banks we explored the online databases AleloVegetal (2022) by the Brazilian Agricultural Research Corporation (EMBRAPA) and Genesys (2022), a Global Portal on Plant Genetic Resources for Food and Agriculture (PGRFA). We also accessed offline data from genebank curators from Instituto Nacional de Tecnología Agropecuária (INTA) of Argentina and Instituto Nacional de Investigación Agropecuaria (INIA) of Uruguay. Thus, passport information of *Solanum malmeanum* from INIA and INTA were obtained personally because they host critical collections of the focused species, although their data are not available at Genesys.

### 2.6 Agronomical traits

To compile the agronomical traits (i.e., biotic resistance, abiotic tolerance levels and quality traits), we explored the online databases Genesys (2022) and literature cited accordingly.

### 2.7 Criteria for updating taxonomic information

We checked and updated the taxonomic determinations of retrieved records of *S. commersonii* and *S. malmeanum* for taxonomic accuracy through the revision of voucher specimens deposited in herbaria or other verifiable sources as genebank pictures of the plants under cultivation. The confirmation, updating or corrections of identifications were possible when the studied specimens matched the current circumscription of *S. malmeanum* based on the observation of diagnostic morphological features present in the physical specimens or in the high-quality digital images retrieved. Accessions cited in previously published works as “*S. commersonii*” and “*S. commersonii* subsp. *malmeanum*” were checked by its code accession at the Germplasm Resources Information Network (GRIN Taxonomy, 2022), to confirm their correct taxonomic identification.

## 3 Results

### 3.1 Taxonomic history, habitat and geographical distribution

Bitter (1913), when describing *S. malmeanum* as a new species to science, emphasized that it can be confused with *S. commersonii* due to their similar habit. Later, Correll (1962) downgrades *S. malmeanum* to a form of *S. commersonii* because he considers it could represent a result of hybridization between *S. commersonii* and *S. chacoense* stating that “*Solanum* form *malmeanum*” differs from the typical form of *S. commersonii* because the former contains 4-5 lateral leaflets pairs and two or more lateral interstitial segments while the typical form of *S. commersonii* contains 2-5 lateral leaflet segments pairs, without or rarely bearing lateral interstitial segments pairs. Another difference on flower characteristic reported by Correll (1962) was that *S. malmeanum* has its corolla narrowly and deeply lobed, often to near the base than *S. commersonii*.

Hawkes and Hjerting (1969) states that Correll was mistaken in classifying *S. malmeanum* as a form of *S. commersonii* because they consider it consists of an assemblage of biotypes with a distinct geographical distribution which are adapted to a habitat range different from that of the typical *S. commersonii.* According to them, *S. malmeanum* has lateral leaflet segments generally decreasing gradually to the leaf base and are narrowly decurrent, being normally petiolulate, while the inflorescence peduncle is unbranched or has the branches not markedly contracted with pedicels articulation median to higher than in *S. commersonii*, and corollas are always white and the lobes about as long as broad. Conversely, *S. commersonii* shows lateral leaflet segments decreasing rapidly in size towards the base of the leaf, being often markedly decurrent and normally sessile, while the inflorescence peduncle branches and is somewhat contracted with pedicels articulation lower to median, and corollas are generally tinged with purple and have the lobes about 1 and a ½ time as long as broad or even longer than in *S. malmeanum*. The two authors reinforced that the two taxa could almost be considered to differ from each other at the species level.

The last comprehensive taxonomic treatment including *S. malmeanum* was by Spooner and collaborators (2016). According to them, *S. malmeanum* can be differentiated from *S. commersonii* by its generally subequal uppermost lateral leaflets that do not decrease rapidly in size towards the leaf base, by its generally petiolulate and larger lateral interstitial segments and by its white corollas. The corollas are almost white, although in a very few cases they have observed a small brushstroke on the abaxial midribs of the corolla lobes very different from the characteristic dark stripes along the abaxial midribs of the corolla lobes in flowers of *S. commersonii*. Maybe, these specimens could represent hybrids between *S. malmeanum* and *S. commersonii*.

*Solanum malmeanum* is native to the Southern Cone of South America, in Argentina, Brazil, Paraguay and Uruguay. It is found in diverse environments such as shady forests, grasslands, damp pastures, roadsides, stream sides, as well as invasive at crop cultivations, including potato fields (Hawkes and Hjerting, 1969). In Argentina it is found in the Eastern Subtropical Biogeographic Province at grasslands or savannas and the Mixed Forest Biogeographic Districts, Chaco Biogeographic Province by Eastern Chaco District (more humid eastern parts) and along the Espiñal Biogeographic Province by Santa Fé and Entre Ríos (Hawkes and Hjerting, 1969). In Brazil, it occurs in the *Araucaria angustifolia* subtropical Mixed Forests of the Paranense Biogeographic Province, with high annual rainfall, as well as in the Pampean Biogeographic Province with a dominant vegetation of grasses and bushes (Hawkes and Hjerting, 1969). In Paraguay, it spreads through the Mixed Forest Biogeographic District into the wetter parts of the Chaco Biogeographic Province and the Chaqueño Biogeographic Domain that are characterized by xerophytic vegetation (Hawkes and Hjerting, 1969). In Uruguay it is found mainly in the so-called ‘Galería Uruguayense’, that comprises the forests along the Uruguay river basin, belonging to the Mixed Forest Biogeographic District of the Eastern Subtropical Biogeographic Province and to the Pampean Biogeographic Province, with a dominant vegetation of grasses and bushes (Hawkes and Hjerting, 1969; Morrone et al.,2014; Spooner et al., 2016). Records found in the surveyed herbaria and genebanks range from sea level up to 765 m of elevation (AleloVegetal, 2022; Global Biodiversity Information Facility, 2022).

### 3.2 Herbarium representativeness

**Figure 1:**
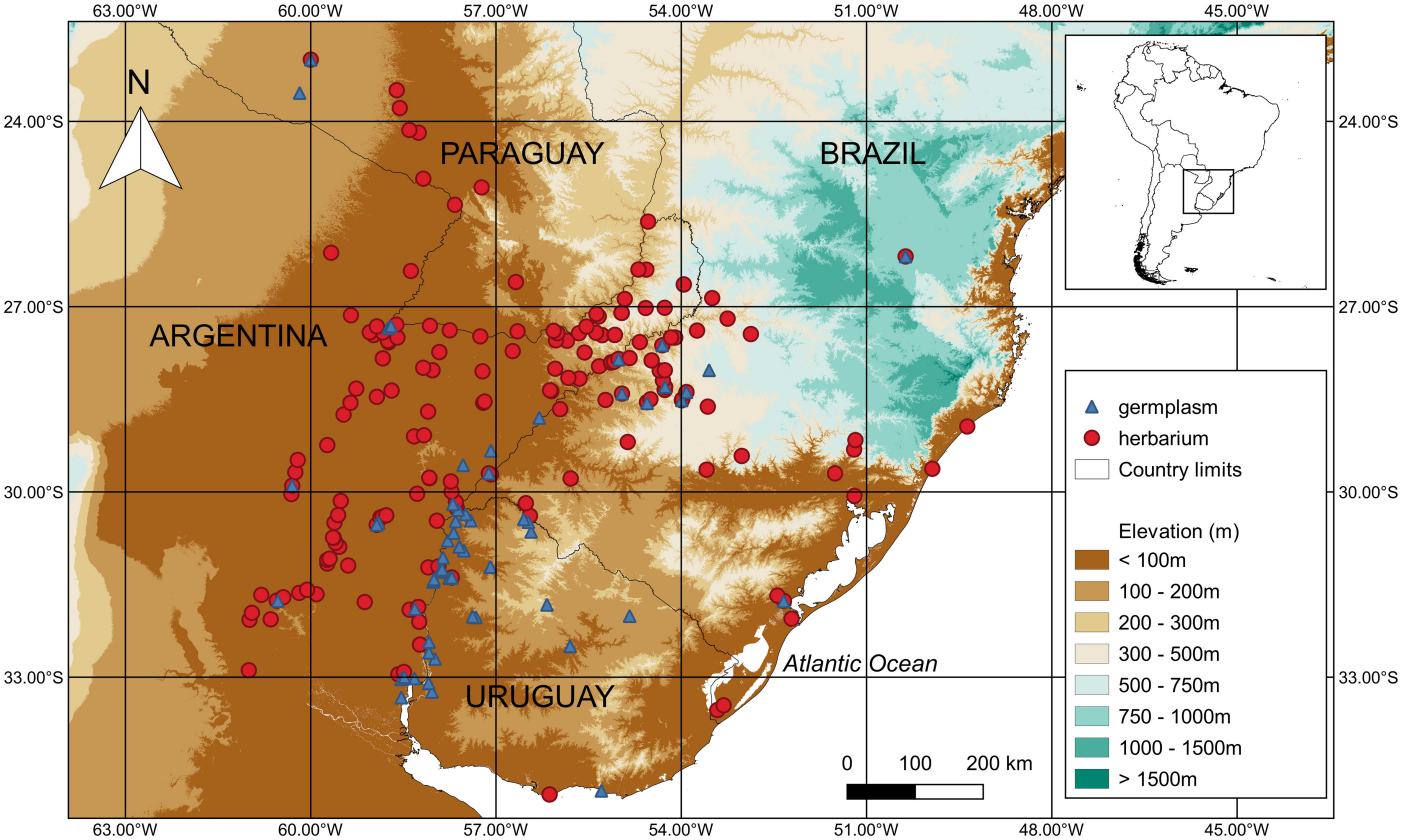
Geographical distribution of *Solanum malmeanum* (Solanaceae) in Southern South America. Occurrence points are represented by red circles for herbarium specimens and blue triangles for accessions in genebanks.

A total of 281 records for *S. malmeanum* specimens are preserved in many herbaria, of which 197 with geographical coordinates were plotted (**Figure 1**).

### 3.3 Genebank representativeness

Currently, *S. malmeanum* is represented in genebanks by a total of 89 accessions worldwide (**Table 1**). The *in vitro* collection at the Instituto Nacional de Investigación Agropecuaria (INIA) Las Brujas, Uruguay in collaboration with Facultad de Agronomía of Universidad de La Republica (Udelar) Uruguay, contains the widest representativeness of *S. malmeanum* conserved ex-situ (36 accessions) followed by U.S. National Plant Germplasm System (NPGS) USDA (25 accessions), and Embrapa Potato Genebank (11 accessions), Brazil. The External Branch North of the Department Genebank of Germany, the Center for Genetic Resources (CGN) of the Netherlands, Instituto Nacional de Tecnología Agropecuária (INTA) of Argentina and the James Hutton Institute (JHI) of United Kingdom account together for a total of 17 accessions.

**Table 1:** Representativeness of *Solanum malmeanum* (Solanaceae) accessions at genebanks (Genesys, 2022; WIEWS, 2022).

Country	Institution/genebank	Genebank code	Number of accessions
Brazil	Brazilian Agricultural Research Corporation (EMBRAPA)	BRA020	11
Netherlands	Centre for Genetic Resources, the Netherlands (CGN)	NDL037	6
Germany	External Branch North of the Department Genebank (IPK)	DEU159	8
Uruguay	Instituto Nacional de Investigación Agropecuaria (INIA)	URY008, URY032	36
Argentina	Instituto Nacional de Tecnología Agropecuaria (INTA)	–	1
United Kingdom	The James Hutton Institute (JHI)	GBR251	2
USA	The U.S. National Plant Germplasm System (NPGS)	USA004	25
	Total		89

A total of 89 collected samples of *S. malmeanum* are ex-situ conserved in genebanks (G) (**Table 2**), whereas only four collected samples are conserved in duplicates (OKADA5071, OKADA7270, OKADA7292, and OKADA7254). From the 89 collected samples, 81 with geographic coordinates are represented in **Figure 1**. For eight collected samples from G it was not possible to retrieve geographic coordinates data.

**Table 2:** Summary of *Solanum malmeanum* (Solanaceae) accessions at genebanks following Country, Collection Code, Accession Code, Latitude and Longitude (Genesys, 2022).

Country	Collection Code	Accession Code	Latitude	Longitude
ARGENTINA	BAL	2015020	-	–
BRAZIL	BRA020	BRA 00183754-1	-31.772	-52.342
BRAZIL	BRA020	BRA 00167436-5	-31.772	-52.343
BRAZIL	BRA020	BRA 00167432-4	-28.563	-54.554
BRAZIL	BRA020	BRA 00167071-0	-28.517	-53.992
BRAZIL	BRA020	BRA 00167433-2	-28.408	-54.961
BRAZIL	BRA020	BRA 00167093-4	-28.299	-54.263
BRAZIL	BRA020	BRA 00183755-8	-27.856	-55.016
BRAZIL	BRA020	BRA 00183756-6	-27.856	-55.016
BRAZIL	BRA020	BRA 00167137-9	-27.626	-54.308
BRAZIL	BRA020	BRA 00183778-0	-26.187	-50.368
BRAZIL	BRA020	BRA 00167394-6	-26.187	-50.368
GERMANY	DEU159	WKS 32184	-33.020	-58.320
GERMANY	DEU159	WKS 35339	-23.540	-60.180
GERMANY	DEU159	WKS 32181	–	–
GERMANY	DEU159	WKS 35341	-28.030	-53.550
GERMANY	DEU159	WKS 35340	-23.000	-60.000
GERMANY	DEU159	AKS 23002	–	–
GERMANY	DEU159	WKS 32182	–	–
GERMANY	DEU159	WKS 32183	–	–
NETHERLANDS	NLD037	CGN22351	-33.331	-58.533
NETHERLANDS	NLD037	CGN22352	-30.533	-58.933
NETHERLANDS	NLD037	CGN18215	-30.200	-57.700
NETHERLANDS	NLD037	CGN21353	-29.700	-57.117
NETHERLANDS	NLD037	CGN18025	-29.567	-57.533
NETHERLANDS	NLD037	CGN18329	-32.500	-55.800
UNITED KINGDOM	GBR251	CPC 7520	–	–
UNITED KINGDOM	GBR251	CPC 7058	–	–
UNITED STATES	USA004	PI 472849	-30.500	-58.900
UNITED STATES	USA004	PI 472843	-33.033	-58.533
UNITED STATES	USA004	PI 472846	-33.033	-58.533
UNITED STATES	USA004	PI 472844	-33.000	-58.483
UNITED STATES	USA004	PI 472840	-31.894	-58.315
UNITED STATES	USA004	PI 472855	-31.766	-60.533
UNITED STATES	USA004	PI 472850	-30.533	-58.933
UNITED STATES	USA004	PI 472854	-30.533	-58.933
UNITED STATES	USA004	PI 472845	-30.200	-57.700
UNITED STATES	USA004	PI 498416	-29.900	-60.300
UNITED STATES	USA004	PI 472847	-29.700	-57.116
UNITED STATES	USA004	PI 458318	-29.566	-57.533
UNITED STATES	USA004	PI 472841	-29.566	-57.533
UNITED STATES	USA004	PI 472851	-29.566	-57.533
UNITED STATES	USA004	PI 472842	-29.333	-57.083
UNITED STATES	USA004	PI 472848	-29.333	-57.083
UNITED STATES	USA004	PI 414154	-28.800	-56.300
UNITED STATES	USA004	PI 472853	-27.350	-58.750
UNITED STATES	USA004	PI 472852	-27.316	-58.700
UNITED STATES	USA004	PI 320269	-28.383	-53.916
UNITED STATES	USA004	PI 498417	-28.383	-53.916
UNITED STATES	USA004	PI 498419	-28.383	-53.916
UNITED STATES	USA004	PI 498408	–	–
UNITED STATES	USA004	PI 498418	-23.000	-60.000
UNITED STATES	USA004	PI 320268	-31.387	-57.716
URUGUAY	INIA	M6P6	-34.840	-55.294
URUGUAY	INIA	RN9P2	-33.239	-58.037
URUGUAY	INIA	RN5P2	-33.098	-58.091
URUGUAY	INIA	RN4P1	-32.705	-57.989
URUGUAY	INIA	RN3P1	-32.604	-58.086
URUGUAY	INIA	P3P2	-32.432	-58.085
URUGUAY	INIA	P3P1	-32.432	-58.085
URUGUAY	INIA	P1P1	-32.032	-57.326
URUGUAY	INIA	P2P2	-32.021	-57.380
URUGUAY	INIA	P2P1	-32.021	-57.380
URUGUAY	INIA	R10P1	-32.011	-54.837
URUGUAY	INIA	T3P2	-31.828	-56.175
URUGUAY	INIA	S1P19	-31.458	-58.020
URUGUAY	INIA	S1P12	-31.456	-58.020
URUGUAY	INIA	S36P1	-31.445	-58.010
URUGUAY	INIA	S35P1	-31.415	-58.005
URUGUAY	INIA	S32P1	-31.396	-57.761
URUGUAY	INIA	S31P1	-31.390	-57.714
URUGUAY	INIA	S39P1	-31.295	-57.867
URUGUAY	INIA	S38P1	-31.272	-57.906
URUGUAY	INIA	S1P1	-31.257	-57.875
URUGUAY	INIA	S5P3	-31.217	-57.091
URUGUAY	INIA	S5P2	-31.217	-57.091
URUGUAY	INIA	S43P1	-31.065	-57.859
URUGUAY	INIA	S43P2	-31.065	-57.859
URUGUAY	INIA	S41P1	-30.951	-57.521
URUGUAY	INIA	S42P1	-30.889	-57.595
URUGUAY	INIA	S40P1	-30.789	-57.783
URUGUAY	INIA	A12P1	-30.665	-57.687
URUGUAY	INIA	A2P3	-30.646	-56.432
URUGUAY	INIA	A6P1	-30.490	-56.475
URUGUAY	INIA	A11P1	-30.478	-57.654
URUGUAY	INIA	A7P2	-30.464	-57.409
URUGUAY	INIA	A3P2	-30.445	-56.551
URUGUAY	INIA	A8P1	-30.364	-57.492
URUGUAY	INIA	A9P2	-30.292	-57.615

### 3.4 Genetic variability characterization

Although there are only a few studies on genetic variability which include accessions from *Solanum malmeanum*, in general, the results point at a close relationship with *S. commersonii* but allow to distinguish both species from each other and from *S. chacoense* (Lester, 1965; Jacobs et al., 2008; Siri et al., 2009). Through immune-electrophoretic analysis, one shared tuber antigen found in *S. malmeanum* and *S. commersonii* was absent from *S. chacoense*, pointing to a closer relationship between these first two species (Lester, 1965).

In order to analyze genetic variability within the species and its relationship with closely related species, molecular markers have been applied among different accessions from *S. malmeanum* across its distribution. Partial sequences of a new retrotransposon *Tnt1* family member, Retrosol, were reported in several *Solanum* sect. *Petota* species, including *S. malmeanum* (Manetti et al., 2007). The accession BAL80009 presented three different fragment sequences of Tnt1 with an average of 423+/-(5) base pairs (bp) and 0.093 πJC nucleotide diversity with Jukes and Cantor (1969) corrections.

Phylogenetic analyses using AFLP markers confirmed that *S. malmeanum* can be genetically separated from *S. chacoense* and *S. commersonii*, because two of 12 accessions of *S. commersonii* that were allocated in the same group with seven *S. malmeanum* accessions (Jacobs et al., 2008), were later confirmed to in fact belong to *S. malmeanum* (CGN 22351, GLKS 35340). Siri et al. (2009) assessed the genetic diversity of *S. commersonii* and *S. malmeanum* from different geographical areas of Uruguay by using different PCR-based markers (three primers for AFLP, 10 primers for RAPD, and four primers for SSR). They found a high consistency of clustering with the geographic origin of accessions, with *S. malmeanum* (cluster B) from the north of Uruguay and *S. commersonii* (cluster A) from the south, previously characterized in morphology (see Siri et al., 2009). When associated with morphological features, the PCR-based markers were able to discriminate *S. malmeanum* from *S. commersonii*. Few studies on the geographic distribution of genetic diversity for *S. commersonii* and *S. malmeanum* are available. In addition, the entire potential area in which both taxa are geographically distributed has not yet been explored, and further studies are needed for a complete and more accurate coverage of the geographic distribution.

### 3.5 Agronomical traits

Many interesting traits have been found in *S. malmeanum* and are summarized in **Table 3**. Most of them are not found in the cultivated potato gene pool, for example, some accessions are resistant against bacterial (*Ralstonia solanacearum* (Smith) Yabuuchi) and verticillium wilt (*Verticillium dahliae* Kleb.); ring rot (*Corynebacterium michiganensis* subsp. *sepedonicus* Spieck. & Kotth.); late (*Phytophthora infestans* Mont.) and early blight (*Alternaria solani* Elis and Martin); fusarium dry rot (*Fusarium sambucinum* Fuckel); hapla (*Meloidogyne hapla* Chitwood) and cyst nematode (*Globodera rostochiensis* Wollenweber); colorado potato beetle (*Leptinotarsa decemlineata* Say); potato leaf hopper (*Empoasca fabae* Harris); green peach aphid (*Myzus persicae* Sulzer); potato aphid (*Macrosiphum euphorbiae* Thomas); and potato leafroll virus (PLRV) (*Polerovirus* sp.) (Radcliffe and Laufr, 1971; Flanders and Radcliffe, 1992; Laferriere et al., 1999; Micheletto et al., 1999; Micheletto et al., 2000; Siri et al., 2009; National Plant Germplasm System, USDA, 2022). *Solanum malmeanum* stands out because of its frost tolerance and ability to cold acclimate at field conditions (i.e., increase cold tolerance after gradually increasing exposure to low temperatures), but also the capacity to be tolerant to heat (Vega and Bamberg, 1995; Genesys, 2022). Valuable quality traits for industrial purposes such as high dry matter, high protein and low reducing sugar content in tubers were also reported (Rocha et al., 2000; Chalá et al., 2001; Jansen et al., 2001) (**Table 4**).

**Table 3:** Resistance against abiotic and biotic stresses reported for the wild potato *Solanum malmeanum* (Solanaceae).

Trait	Agent	Accession code or identification	Source
Abiotic stress	Frost	PI 320269; PI 414154; PI 472840; PI 472841; PI 472841; PI 472843; PI 472844; PI 472845; PI 472846; PI 472847; PI 472848; PI 472849; PI 472851; PI 472852; PI 472853; PI 472854; PI 472855; PI 498408; PI 498416; PI 498417; PI 498418; PI 498419; MLM266-2	(Tu et al., 2021; Genesys, 2022)
	Heat	PI 498416	(Genesys., 2022)
Biotic stress	Bacterial wilt (*R. solanacearum)*	Accession code or identification not provided	(Siri et al., 2009)
	Ring rot (*C. michiganensis* subsp. *sepedonicus*)	PI 458318; PI 472841	(Genesys, 2022)
	Early blight (*A. solanii)*	PI 472840	(Genesys, 2022)
	Late blight (*P. infestans)*	OKA 7256; OKA 7256.01; OKA 7256.07; OKA 7256.08; OKA 7292; OKA 7310; OKA 7310.01	(Micheletto et al., 1999; Micheletto et al., 2000)
		PI 472844; PI 472846; PI 472847; PI 472850; PI 472853; PI 472855	(Genesys, 2022)
	Verticillium wilt (*V. dahliae)*	PI 472851	(Genesys, 2022)
	Green peach aphid (*M. persicae*)	PI 320269; PI 458318; PI 472840	(Radcliffe and Laufr, 1971; Genesys, 2022)
	Potato aphid (*M. euphorbiae*)	PI 320269	(Radcliffe and Laufr, 1971)
	Colorado potato beetle (*L. decemlineata*)	PI 458318	(Flanders and Radcliffe, 1992)
	Potato leafhopper (*E. fabae*)	PI 472851; PI 472843	(Flanders and Radcliffe, 1992)
	Potato cyst nematode (*G. rostochiensis)*	Accession code or identification not provided	(Castelli et al., 2003)
	Root-knot nematode (*M. hapla)*	PI 472841	(Genesys, 2022)
	Potato leaf roll virus (*Polerovirus* sp.)	PI 458318	(Genesys, 2022)

**Table 4:** Quality traits found in potato wild relative *Solanum malmeanum* (Solanaceae) accessions.

Organ	Specificities	Accession	References
Tubers	Dry matter content 28.6% – 34.62%	CL55=BRA 00167071-0; CL57=BRA 00167093-4; CL60=BRA 00183755-8; CL63=BRA 00183756-6; CL65=BRA 00167137-9	(Rocha et al., 2000; Jansen, 2001; AleloVegetal, 2022)
	Tuber starch 15.4 – 23%	Accession code or identification not provided	(Jansen, 2001)
	Starch granules size 45.8 um	Accession code or identification not provided	(Jansen, 2001)
	Reducing sugar 0.19 –0.33%	CL63= BRA 00183756-6; CL65= BRA 00167137-9	(Chalá et al., 2001; Alelo Vegetal, 2022)
	Protein 4.5% of dry matter	Accession code or identification not provided	(Jansen et al., 2001)
	Amylose 32.1% of amylose content in starch	Accession code or identification not provided	(Jansen et al., 2001)
Leaves	Glycoalkaloid (dehydrocommersonine) 23%	PI 320269	(Gregory et al., (1981)

### 3.6 *Solanum malmeanum* as a source of traits for the generation of new potato cultivars

#### 3.6.1 Frost and heat tolerance

*Solanum malmeanum* is particularly interesting due to its freezing tolerance and great capacity to cold acclimate (i.e., increase cold tolerance after exposure to low, non-freezing temperatures) (Hawkes, 1958; Hudson, 1961; Ross and Rowe, 1965; Hawkes and Hjerting, 1969; National Plant Germplasm System, USDA, 2022; Genesys, 2022). Twenty-three accessions were reported to be frost resistant and one accession is also reported to be moderately resistant to heat stress (**Table 3**). It has been reported to be capable of surviving at extremely low temperatures (–0.55°C to –5°C), with none or a relatively small percentage (0–20%) of the leaf area damaged (Ross and Rowe, 1965; Hawkes and Hjerting, 1969; Vega and Bamberg, 1995). Earlier observations reported that wild potatoes that have a rosette habit, such as *S. malmeanum*, generally are more highly resistant to cold and frost (Firbas and Ross, 1962). Because most potato cultivars are sensitive to low temperatures and are unable to cold acclimate at temperatures lower than –3°C (Chen and Li, 1980), great efforts are carried out to find sources of tolerance and to introgress this trait into cultivated potato. A *Solanum malmeanum* freezing tolerant accession (MLM266-2) was fused with freezing susceptible dihaploid *S. tuberosum* (AC142) by somatic hybridization. Shoots were regenerated from calli formed, excised and cultured on MS medium (Tu et al., 2021). Somatic hybrids submitted to a correlation analysis between freezing tolerance and tuberization capacity of progenies generated from the first backcrossing with tetraploid cultivars *S. tuberosum* indicated that these traits are controlled by independent genetic loci (Tu et al., 2021).

#### 3.6.2 Biotic stresses

##### 3.6.2.1 Bacterial

###### 3.6.2.1.1 Bacterial wilt

In the range of geographical distribution of *Solanum malmeanum* in Southern South America, the predominant *R. solanacearum* belong to phylotype II sequevars (Siri et al, 2009; Santiago et al., 2017). *Solanum malmeanum* is documented to possess interesting levels of resistance against *R. solanacearum* phylotype II sequevars 1-2 previously isolated from different potato fields in Uruguay (Siri et al., 2009), evaluated under controlled conditions. Two accessions were classified to be resistant (e.g. absence of wilting symptoms) and three accessions demonstrate moderate resistance. Resistant accessions are summarized in **Table 3**. Little is known about the resistance across the range of distribution, however, there are ongoing studies aiming at characterizing resistance levels of potato wild species accessions across the distribution in Uruguay. Considering that each strain of bacterial wilt is present at specific geographical regions, the knowledge of its variability and diversity could be an efficient guide to identify new potential sources of resistance from different geographical populations.

###### 3.6.2.1.2 Bacterial ring rot

Two accessions (PI 458318 and PI 472841) proved to be resistant against bacterial ring rot of potato (**Table 3**) (Genesys, 2022; National Plant Germplasm System, USDA, 2022). Ring rot is caused by *Corynebacterium michiganensis* subsp. *sepedonicus* (Spieckermann & Kotthoff).

##### 3.6.2.2 Fungal

###### 3.6.2.2.1 Potato early blight

The genotype PI 472840 from Argentina was evaluated and has been reported to be resistant against potato early blight (**Table 3**) (Wolters et al., 2021; Genesys, 2022; National Plant Germplasm System, USDA, 2022). Genotype MLM 266-2 was evaluated under greenhouse conditions and showed no signs of infection, but small dark spots occurred due to the hypersensitive response (Wolters et al., 2021). MLM 266-2 was crossed with diploid *S. tuberosum*, producing a triploid progeny with high levels of resistance inherited dominantly (Wolters et al., 2021). The disease is caused by *Alternaria solani* (Ellis. & G. Martin) Ser., a leaf-spotting and defoliation agent that is responsible for significantly reduced yields in many tropical and subtropical producing regions (Rotem, 1994; Xue et al., 2022).

###### 3.6.2.2.2 Potato late blight

In total thirteen accessions were reported to be resistant to late blight (**Table 3**). The genotypes OKA 7310.01, OKA 7256.08, OKA 7256.07, OKA 7256.01 showed an incompatible reaction after inoculation with R0 in trials under greenhouse conditions. A second experiment aimed to reconfirm the presence of *R*-genes and the offspring produced by the cross between OKA 7310.01 (resistant to R0) and OKA 7282.06 (susceptible to R0) was inoculated with a complex race of late blight. Among the offspring of 300 plants, 70% (210) showed incompatible (resistance) responses, confirming the presence of the *R* genes in *S. malmeanum* (Micheletto et al., 1999; Micheletto et al., 2000). Micheletto and collaborators (2000) screened four Argentinean diploid accessions for quantitative resistance to late blight *Phytophthora infestans* virulent (R1, R3, R4, R5, R7, R10, R11) and non-virulent (R0) complex races under both greenhouse and field conditions during two seasons (1996/1997 and 1997/98) to assess the year × genotype interaction. Results showed high variability reaction of *P. infestans* among genotypes. Two accessions were susceptible (OKA 7282, OKA 7291), while two resistant accessions segregated for resistance (**Table 3**). OKA 7292 genotypes 5 and 9 and OKA 7256 genotypes 2, 7 and 9 performed as resistant. Furthermore, no genotype × year interaction was detected, and the behavior was consistent across years. Although Micheletto and collaborators (1999; 2000) treat the evaluated accessions as *S. commersonii*, they are currently to be considered as *S. malmeanum* (Spooner et al., 2014; Spooner et al., 2016; Global Biodiversity Information Facility, 2022). The disease caused by the oomycete *Phytophthora infestans* (Mont.) de Bary is considered the most important disease of potato crop in all producing regions worldwide (Santana et al., 2013; Göre et al., 2019).

###### 3.6.2.2.3 Verticillium wilt

The accession PI 472850 is reported to be immune against *Verticillium dahliae* (**Table 3**) (National Plant Germplasm System, USDA, 2022). Verticillium wilt is one of the most important soilborne fungal diseases on potato, also known as potato early dying disease, caused by *V. dahliae* Klebahn and *V. albo-atrum* Reinke & Berthold (Frost et al., 2007; Simko and Haynes, 2017; Li et al., 2019).

###### 3.6.2.2.4 Fusarium dry rot

One accession (PI 414154) was screened in a tuber disk essay for resistance to *Fusarium sambucinum* (Lynch et al., 2003), however it responded as susceptible. Fusarium dry rot of potato tubers is caused by many species such as *F. avenaceum*, *F. culmorum*, *F. equiseti*, *F. oxysporum*, *F. sambucinum*, *F. solani*, among others. It is a severe disease responsible for causing significant economic losses mainly during the post-harvest and storage periods (Peters et al., 2008; Du et al., 2012; Gachango et al., 2012).

###### 3.6.2.2.5 Nematodes

*Solanum malmeanum* showed full resistance to potato cyst nematode *Globodera rostochiensis* and susceptibility to *G. pallida*, but the inheritance of resistance was not investigated (Castelli et al., 2003). Furthermore, it has also been reported to be resistant against root-knot nematode (*Meloidogyne hapla*) (**Table 3**) (National Plant Germplasm System, USDA, 2022). Nematodes, when the population density is high, negatively affect potato production from the field, restricting plant growth, to the post-harvest period, rendering tubers unmarketable (Contina et al., 2019; Koirala et al., 2020).

###### 3.6.2.2.6 Insect pests

*Solanum malmeanum* was moderately resistant against potato leafhopper (*Empoasca fabae* Harris), although the defense mechanisms have not been elucidated, as well as fully resistant to potato aphid *Macrosiphum euphorbiae* (Thomas) and potato green peach aphid *Myzus persicae* (Sulzer) (**Table 3**) (Radcliffe and Laufr, 1971; Genesys, 2022; National Plant Germplasm System, USDA, 2022). Insect resistance in potato is mainly associated to morphological characteristics, density on leaves and exudates of secondary metabolites of glandular trichomes, which provide protection against herbivory (Flanders et al., 1992). Also, an important natural source of insect resistance in potato is related to the production of high levels of foliar glycoalkaloids (Coombs et al., 2002). Some results suggest that this is an important mechanism of insect resistance found in *S. malmeanum*.

#### 3.6.3 Viruses

Accession PI 458318 from Argentina has been reported to be resistant against potato leaf roll virus (*Polerovirus* sp.) (**Table 3**) (Genesys, 2022; National Plant Germplasm System, USDA, 2022). *Solanum malmeanum* has also been reported to be susceptible and spontaneously infected by the systematic ringspot virus (TMV tobacco mosaic virus) that is transmitted from generation to generation *via* tubers (Hansen, 1960).

#### 3.6.4 Quality traits for production and industry

##### 3.6.4.1 Tuber dry matter, starch and protein contents

*Solanum malmeanum* has been reported (**Table 4**) to contain a range from 28.6% to 34.62% of dry matter content (Rocha et al., 2000; Jansen et al., 2001), 15.4 to 23% starch content (Jansen et al., 2001), 4.5% of protein in tuber dry matter content and 32.1% of amylose content in starch, with a mean particle diameter of starch granules of 45.8 μm (CV%=9.2) (Jansen et al., 2001). In addition, reducing sugars on its tubers have been evaluated from two different growing seasons, with an average of 0.22% in spring, and 0.33% in autumn, far below the levels presented in the reference cultivar ‘Baronesa’ (*S. tuberosum*) (Chala et al., 2001). A strong association in diploid *S. malmeanum* clones was verified between glutamate oxaloacetate transaminase (GOT) at relative mobility band 1.00 of electrophoretic standards and high dry matter content (average of 34.62 of dry matter content on tubers). The use of the GOT enzyme may be efficient in more accurately predicting this character in the early stages of selection in diploid than tetraploid species (Andreu and Pereira, 2004).

##### 3.6.4.2 Glycoalkaloids

Steroidal glycoalkaloids are secondary metabolites toxic for human consumption, although they may also have advantages to improve resistance against viral, insect-pests or microbial diseases. *Solanum* ser. *Commersoniana* species mainly produces α-solanine and β-chaconine (Schreiber, 1963). *Solanum malmeanum* accession PI 320269 (cited as *S. commersonii*) has been reported to contain 23% (mg/g dry leaves) of glycoalkaloid content on leaves (**Table 4**), where 100% is dehydrocommersonine, with undetectable levels (<3%) of α-solanine and β-chaconine (Gregory et al., 1981) (**Table 4**). Dehydrocommersonine was associated in *Solanum oplocense* Hawkes with host resistance against *Leptinotarsa decemlineata* (Tai et al., 2014; Tai et al., 2015), then *S. malmeanum* could be predicted as a potential source of resistance against this pest. There is room for selection because they are controlled by genetic factors (Mensinga et al., 2005) and even though high levels of foliar steroidal glycoalkaloids are frequently associated with high contents in tubers (Sanford et al., 1992), they are accumulated during the development of each organ (Krits et al., 2007; Mweetwa et al., 2012; Paudel et al., 2019; Zhen et al., 2019). This differential accumulation behavior allows to change the distribution of accumulation in specific plant tissues across some generations of selection (Sanford et al., 1992; Umemoto et al., 2016; Nahar et al., 2017; Zhen et al., 2019), so it should be possible to select accessions from *S. malmeanum* for breeding to obtain cultivars with resistance against pests given by high foliar glycoalkaloids content and with low toxicity for human consumption due to low levels of glycoalkaloids in the tubers.

### 3.7 Cytogenetics

*Solanum malmeanum* is a diploid species (2n = 2x = 12) with occasional triploid plants (Hawkes and Hjerting, 1969; Tarn and Hawkes, 1986; Spooner et al., 2016). Diploid plants usually produce higher frequency of stainable pollen than triploids. The mean pollen diameter in equatorial view is 23 µm in diploid plants and varies from 18.5 to 24.9 µm in triploids (Tarn and Hawkes, 1986).

Some accessions of *S. malmeanum* showed regular meiotic abnormalities observed at microsporogenesis, including chromosomes out of the equatorial plate in metaphases I and II (MI and MII) and lagging chromosomes in anaphases I and II, and telophases I and II (Pandolfi, 1998; Tomé et al., 2007; Tomé et al., 2009). Furthermore, a range from 3.7% to 50.8% of pollen mother cells (PMC) with meiosis abnormalities was observed. The frequency of abnormal PMC decreased in the later stages of meiosis. This, together with a good pollen viability (60.33 – 96.5%), suggests the existence of selection against abnormal PMC cells during the microsporogenesis process with implications for the choice of pollen donors for crosses.

Pollen viability was significantly higher in *S. malmeanum* than in cultivated potatoes (Pandolfi, 1998; Tomé et al., 2007). It also produces a high pollen amount, ranging from 454.20 to 476.20 pollen grains per field, exceeding the means of *S. tuberosum* clones (Tomé et al., 2007). According to Hardigan et al. (2017), the reduction of pollen viability of cultivated potato (*S. tuberosum*) is a consequence of the domestication syndrome. Although the accessions SCM57 (BGB017) and SCM60 (BGB447) (AleloVegetal, 2022) did not show unreduced pollen grains, they had meiotic mechanisms (parallel spindles) that possibly can lead to the formation of unreduced pollen, presenting the highest percentages of cells with parallel spindles (27% and 22%). Furthermore, pollen grain size of *S. commersonii* (13.68–35.52 µm) tends to be larger than that of *S. malmeanum* (11.4 to 23.9 µm) (Tomé et al., 2009). This approach put forward by Tomé et al., (2009) raises the hypothesis that *S. malmeanum* has no pollen grain diameters satisfying the value of 25 µm suggested by Quin et al. (1974) and Ramanna (1974) and would imply the proposition of a new threshold for unreduced (2n) pollen grains. However, the selection of 2n pollen based only on diameter and shape of the pollen grain is feasible only in species with known differences between haploid and diploid pollen grains (Parrott and Smith, 1984; Parrott and Smith, 1986). According to Ordoñez et al. (2017), 2n pollen have a diameter 1.2 times larger than reduced gametes (n). 2n gametes production can be predicted by crossing the species of interest with another different EBN group species, and then observing pollen tube growth, fruit set, seed set and germination success. Two of 12 clones of *S. malmeanum* (PI 414154, cited as “*S. commersonii*”) were reported to produce 2n pollen (den Nijs and Peloquin, 1977a). Therefore, it is expected that in natural populations, functional 2n gametes can be found.

### 3.8 Reproductive biology, breeding system and endosperm balance number

*Solanum malmeanum* is a tertiary gene pool (1EBN) wild relative of potato and is considered reproductively isolated from other tuber-bearing 2EBN and 4EBN species (Hanneman, 1994; Spooner et al., 2016). *Solanum malmeanum* reproduces vegetatively by tubers and stolons and sexually by botanical seeds. From five diploid accessions, four were characterized as self-incompatible (Nicolao, 2021) and one self-compatible accession of *S. malmeanum* was found (Nicolao, 2021).

Hanneman (1994) used a diploid female plant of *S. malmeanum* for the assignment of EBN in crossing tests. Although only one accession was used as female to determine its EBN, it produced an average of six seeds per fruit from any crosses with male standard species 2x (1EBN), less than one seed per fruit from 2x (2EBN) species, and no seed set from 4x (4EBN) crosses. Therefore, it was designated as 1EBN.

Crosses between different potato species with the same EBN number, regardless of ploidy, are usually successful (Johnston et al., 1980; Johnston and Hanneman, 1982). Theoretically, species with the same EBN may be intercrossed in nature if sympatry and synchronized flowering occur and pre-zygotic barriers are absent. Tarn and Hawkes (1986) obtained vigorous and fertile hybrid progenies from reciprocal crosses between 2x *S. malmeanum* and 2x *S. commersonii*. The seed production per berry and mean weight per 100 seeds were equally variable. *Solanum commersonii* produces fewer (66.5), but heavier seeds (52.8 mg per 100 seeds) per berry than *S. malmeanum* (128.4 seeds per berry, with 12.8 mg per 100 seeds) (Tarn and Hawkes, 1986). Furthermore, the seedlings grown from reciprocal crosses between *S. commersonii* and *S. malmeanum* usually were vigorous, fertile, highly variable morphologically and generally displayed intermediate morphology between the two parental species (Tarn and Hawkes, 1986). It is interesting to note that parental traits as flower pigmentation (typically purple-pigmented corolla in *S. commersonii* and white (not-pigmented) corolla in *S. malmeanum*), always segregate in F1 progenies with intermediate phenotypes (Tarn and Hawkes, 1986).

Hawkes and Hjerting (1969) made significant efforts to perform reciprocal crosses with *S. chacoense* and obtained only one seed that germinated and generated a vigorous plant. Ehlenfeldt and Hanneman (1988) produced a small number of seeds from many hand pollinations between *S. malmeanum* (accession PI 320269, cited as “*S. commersonii*”) (1EBN) and *S. chacoense* (2EBN) (Ehlenfeldt and Hanneman, 1988). Obtaining hybrid offspring of *S. malmeanum* × *S. chacoense* was possible, but the endosperm barrier between these two species greatly limits viable seed development. In reciprocal backcrosses of the F1 hybrids (1 ½ EBN) with both *S. chacoense* (2EBN) and *S. malmeanum* (1EBN), the results were consistent with a directional effect determining the difference in the ratio of viable seeds to aborted seeds, related to which parent is male and which parent is female in any given cross, but it does not seem to be a cytoplasmic effect, because the same female may produce different results depending on pollen source. This effect is most likely due to the dose-effect in the endosperm (Ehlenfeldt and Hanneman, 1988).

Crossability barriers between 2x 2EBN *S. chacoense* and 2x 1EBN *S. malmeanum* are well-established, but no sexual barriers were observed between 2x 1EBN *S. commersonii* and 2x 1EBN *S. malmeanum*, elucidating that EBN is a strong internal post-zygotic barrier (Johnston et al., 1980; Summers and Grun, 1981; Hanneman, 1994). Thus, it may predict that *S. malmeanum* could freely cross with other 1EBN sympatric wild potatoes such as 2x 1EBN *S. commersonii*, but not with 2EBN such as *S. chacoense*, except when 2n gametes are present (Hawkes and Hjerting, 1969; Hanneman, 1994). Thus, the mechanisms involved in keeping the two species appart in sympatric zones where *S. commersonii* and *S. malmenum* occur still deserve further explanations.

## 4 Discussion

*Solanum malmeanum* is a valuable genetic resource for many traits of importance to the potato industry. This 1EBN wild potato contains genes to combat pests and diseases which are lacking in the primary genepool of potato. However, using this 1EBN wild species to improve cultivated potatoes is challenging. *S. malmeanum* is cross-incompatible with cultivated potatoes due to the strong EBN post-zygotic barrier (Hanneman, 1994). To capture genetic diversity from 1EBN potato species into the cultivated gene pool, breeders have efficiently used the 2x 2EBN Mexican potato species *Solanum verrucosum* as bridge species (Jansky and Hamernik, 2009). Bamberg and collaborators (2021) show the ability of female parent *S. verrucosum* (2EBN) to form interspecific hybrids with 1EBN species as male parent without stylar barrier. Much like *S. verrucosum*, the lack of stylar barriers in cultivated tomato has allowed tomato breeders to access genetically distant wild relatives, even those of different effective ploidy (Fulton et al., 1997; Roth et al., 2019; Städler et al., 2021). Thus, a combination of strategies such as ploidy manipulation, somatic fusion, embryo rescue and transgenesis are expected not to be required to access the valuable 1EBN potato wild species genetic diversity.

Ploidy manipulation methods were established to introgress genetic diversity from diploids to tetraploids (Hermundstad and Peloquin, 1985a; Peloquin et al., 1989a; Jansky et al., 1990; Werner and Peloquin, 1991a; Carputo et al., 2003). Breeders usually use di-haploids (2x 2EBN) derived from crossing tetraploid *S. tuberosum* (Group Tuberosum or Group Phureja) with haploid inducers to enable intercrossing with wild diploid species (Hermundstad and Peloquin, 1985a; Peloquin et al., 1989a; Carputo et al., 2003), or anti-mitotic substances, such as colchicine and oryzalin, to increase the ploidy level of diploid species (Tomé et al., 2016). Diploid F1 offspring that produces 2n gametes are valuable genetic material for capturing allelic diversity from most of the diploid wild potatoes (Hermundstad and Peloquin, 1985a; Peloquin et al., 1989a; Jansky et al., 1990; Jansky and Peloquin, 2006). After many cycles of selection for adaptation and desired agronomic traits, superior diploid hybrids can be crossed with meiotic mutants producing 2n gametes to generate 4x progenies (Peloquin et al., 1989a; Peloquin et al., 1989b; Werner and Peloquin, 1991a; Masuelli et al., 1992; Carputo et al., 2003). Nodal segments of diploid *S. malmeanum* when treated with oryzalin (10-50µM for 24h) successfully induced tetraploid plants (Tomé et al., 2016).

Recently, diploid F1-hybrid breeding has increased attention of breeders due to the possibility of redomestication of potato crop by creating inbred lines from clones carrying mutations of the *Sli* self-incompatibility gene locus (Lindhout et al., 2011; Jansky et al., 2016; Eggers et al., 2021). Diploid inbred lines are outstanding materials for breeding and functional genetics (Marand et al., 2019; Jayakody et al., 2022), because they allow high throughput phenotyping and efficient QTL detection (Manrique‐Carpintero et al., 2015; Hara-Skrzypiec et al., 2018), accelerating the breeding process. An obstacle still present in diploid potato breeding is the high level of inbreeding depression (De Jong and Rowe, 1971; Zhang et al., 2019).

The taxonomic history of *S. malmeanum* can hamper the exploration of its true potential for potato breeding. *Solanum malmeanum* has been genetically understudied compared to *S. chacoense* and *S. commersonii* that have their genomes sequenced (Aversano et al., 2015; Leisner et al., 2018). The utilization of *S. chacoense* is well established in diploid potato breeding programs (Lindhout et al., 2011; Jansky et al., 2014). *Solanum commersonii* is highly exploited mainly for its frost tolerance (Cardi et al., 1993; Esposito et al., 2020; Esposito et al., 2021) and bacterial wilt (*Ralstonia solanacearum*) resistance (Kim-Lee et al., 2005; Siri et al., 2009; Andino et al., 2022).

The morphological variability, wide geographic distribution, diverse array of habitats occupied and ecological preferences of *S. malmeanum* are not fully represented in current genebanks. Limited studies were performed to assess the genetic variability and the few available ones do not fully cover these aspects. Important agronomical traits were documented, although comprehensive studies on this species are lacking and most of the references available for response to abiotic and biotic stresses (Flanders and Radcliffe, 1992; Micheletto et al., 1999; Micheletto et al., 2000; Castelli et al., 2003) and quality traits for production and industry (Rocha et al., 2000; Chalá et al., 2001; Jansen, 2001) are from occasional works or have evaluated *S. malmeanum* as “*S. commersonii*”.

Next Generation Sequencing (NGS) technologies will allow a better understanding of the evolutionary relationships between *S. malmeanum* and *S. commersonii* to provide the required framework for conserving and using this potato wild genetic diversity (Fielder et al., 2015; Dempewolf et al., 2017). The reinstatement at the species level brings new light to a comeback and promising future for the applied uses of this wild potato in classic and cutting-edge techniques for potato breeding. Towards the true exploitation of this promising potato genetic resource, the current taxonomy resulting from the latest treatment must be updated in all genebanks and reference collections to allow better use of the conserved germplasm and enhance its applied uses. Furthermore, with the rise of new technologies, new studies are needed to evaluate the entire geographical distribution of *S. malmeanum* and compare to the other two sympatric potato wild species *S. chacoense* and *S. commersonii*.

Plant breeders require genetic variability information to speed up the search of each desirable agronomic trait, tolerance against abiotic stresses, as well as for the genes encoding for quality traits (Bethke et al., 2017; Swarup et al., 2021).

As a final remark, we highlight that the potential of *S. malmeanum* for potato breeding has been neglected, especially when compared to other close potato wild relatives such as *S. chacoense* and *S. commersonii* (Aversano et al., 2015; Leisner et al., 2018) and must be properly addressed with contemporary molecular tools to unlock its applied use as a promising potato wild relative to face the challenges imposed to the potato crop in the 21^st^ century.

## Author contributions

RN discussed the original idea, reviewed and checked literature data, compiled and wrote the manuscript. PG wrote partially, provided data on the Uruguayan references, and reviewed the text. CC provided advisory, wrote partially and reviewed the text. GH discussed the original idea, provided advisory, wrote partially and reviewed the text. All authors contributed to the article and approved the submitted version.
